# Converting antimicrobial into targeting peptides reveals key features governing protein import into mitochondria and chloroplasts

**DOI:** 10.1016/j.xplc.2023.100555

**Published:** 2023-02-02

**Authors:** Oliver D. Caspari, Clotilde Garrido, Chris O. Law, Yves Choquet, Francis-André Wollman, Ingrid Lafontaine

**Affiliations:** 1 UMR7141 (CNRS/Sorbonne Université), Institut de Biologie Physico-Chimique, 13 Rue Pierre et Marie Curie, 75005 Paris, France; 2 Centre for Microscopy and Cellular Imaging, Biology Department Loyola Campus of Concordia University, 7141 Sherbrooke W., Montréal, QC H4B 1R6, Canada

**Keywords:** antimicrobial peptide, *Chlamydomonas reinhardtii*, endosymbiotic organellogenesis, presequence, targeting peptide, transit peptide

## Abstract

We asked what peptide features govern targeting to the mitochondria versus the chloroplast, using antimicrobial peptides as a starting point. This approach was inspired by the endosymbiotic hypothesis that organelle-targeting peptides derive from antimicrobial amphipathic peptides delivered by the host cell, to which organelle progenitors became resistant. To explore the molecular changes required to convert antimicrobial into targeting peptides, we expressed a set of 13 antimicrobial peptides in *Chlamydomonas reinhardtii*. Peptides were systematically modified to test distinctive features of mitochondrion- and chloroplast-targeting peptides, and we assessed their targeting potential by following the intracellular localization and maturation of a Venus fluorescent reporter used as a cargo protein. Mitochondrial targeting can be achieved by some unmodified antimicrobial peptide sequences. Targeting to both organelles is improved by replacing lysines with arginines. Chloroplast targeting is enabled by the presence of flanking unstructured sequences, additional constraints consistent with chloroplast endosymbiosis having occurred in a cell that already contained mitochondria. If indeed targeting peptides evolved from antimicrobial peptides, then required modifications imply a temporal evolutionary scenario with an early exchange of cationic residues and a late acquisition of chloroplast-specific motifs.

## Teaser

This study shows that amphipathic helical antimicrobial peptides can be used to target proteins to mitochondria, and chloroplast targeting is enabled by unstructured flanking sequences, with helix properties contributing to differential targeting. Targeting is enhanced by addition of a cleavage site and by replacing lysines with arginines. These findings regarding protein import into endosymbiotic organelles are discussed from a mechanistic and evolutionary perspective.

## Introduction

Mitochondria and chloroplasts arose through endosymbiosis and retain their own genomes, but the vast majority of organellar proteins are encoded in the nucleus, translated in the cytoplasm, and imported into the organelles (Chotewutmontri et al., 2017; Wiedemann and Pfanner, 2017). N-terminal targeting peptides (TPs) that are cleaved off upon import provide the information on targeting, although their primary structures are very diverse (Bruce, 2001). Yet chloroplast transit peptides (cTPs) and mitochondrial presequences (mTPs) have very similar physico-chemical properties, often making reliable differentiation challenging. Many studies have found sequence elements contributing to specificity determination, and prediction programs have been improving (Tardif et al., 2012; Armenteros et al., 2019), but a mechanistic understanding of how targeting information is encoded has remained elusive.

Here, we use antimicrobial peptides (AMPs) as an original chassis to test the contribution of key TP features toward targeting efficiency and specificity. Part of the innate immune system, AMPs are produced by virtually all types of cells in a bid to kill or control microbial adversaries (Joo et al., 2016; Lazzaro et al., 2020). AMPs have recently been proposed to be at the evolutionary origin of TPs (Wollman, 2016; Caspari and Lafontaine, 2021). The proposed evolutionary scenario posits that early during endosymbiotic organellogenesis of first the mitochondrion and later the chloroplast, the host cell used AMPs to attack the bacterial proto-endosymbiont. A bacterial resistance mechanism whereby the AMP is imported into the bacterial cytoplasm would have generated a pathway for host proteins to reach the bacterial cytosol—a plausible first step in the evolution of a protein import machinery. Cationic, Helical Amphipathic Ribosomally produced AMPs (HA-RAMPs) and TPs share key physico-chemical properties and have been shown, in some instances, to retain cross-functionality (Garrido et al., 2020): several TPs have antimicrobial activity, and selected HA-RAMPs, fused to a cleavage-site-containing TP element, were shown to promote the import of a Venus fluorescent protein into either the mitochondria or the chloroplast of the model green alga *Chlamydomonas reinhardtii*.

The main part of mTPs and the central element of cTPs most closely resemble HA-RAMPs on account of a shared cationic, amphipathic helical structure that often encompasses the entire length of HA-RAMPs (Caspari and Lafontaine, 2021) and mTPs (von Heijne, 1986; von Heijne et al., 1989). Although plant cTPs have been described as unstructured (von Heijne and Nishikawa, 1991), algal cTPs more closely resemble mTPs in being helical (Franzén et al., 1990). Helices have been observed by NMR in membrane-mimetic environments in algal and plant cTPs (Lancelin et al., 1996; Bruce, 1998, 2001; Krimm et al., 1999; Wienk et al., 2000), and signatures of amphipathic helices can be detected in a majority of cTPs (Garrido et al., 2020).

In addition to the helices, mTPs and cTPs contain recognition sites at the C-terminus, where processing peptidases in the mitochondrial matrix (MPP) and the chloroplast stroma (SPP) cleave off the cargo protein (Teixeira and Glaser, 2013). These recognition sites encompass some 10 residues upstream of the cleavage site and are structurally distinct from the rest of the TPs, even showing a weak sequence conservation (von Heijne et al., 1989; Tardif et al., 2012; Köhler et al., 2015). Although targeting information is usually contained within mTP sequences upstream of the cleavage site, targeting by cTPs shorter than approximately 60 amino acids often requires downstream unstructured sequence stretches in the N-terminal domain of the mature protein (Bionda et al., 2010; Caspari, 2022). Besides the amphipathic helical module and the C-terminal cleavage module shared between mTPs and cTPs, it has been argued that distinct features at their N-termini confer organelle specificity to each set of TPs (von Heijne et al., 1989; Ivey and Bruce, 2000; Ivey et al., 2000; Bhushan et al., 2006; Chotewutmontri et al., 2012; Chotewutmontri and Bruce, 2015; Köhler et al., 2015; Chotewutmontri et al., 2017; Lee et al., 2019).

In this study, we systematically introduced modifications into diverse HA-RAMPs in a bid to generate targeting to the mitochondria or chloroplast in *Chlamydomonas*. This dataset provides new insights into how different TP elements contribute to differential targeting and to the efficiency of protein import. Being similar in physico-chemical properties to TPs (Garrido et al., 2020), HA-RAMPs provide a privileged vantage point from which to study sequence elements that govern targeting. In our choice of HA-RAMPs, we aimed to reflect the diversity of available sequences by choosing representatives of different HA-RAMP families based on similarity with TPs. We show that some of our 13 HA-RAMPs natively contain TP-like sequence elements, with some HA-RAMPs being prone to chloroplast targeting and others showing a preference for the mitochondria. Furthermore, we provide evidence for a critical functional difference in cationic residues, with lysine (K) being used in HA-RAMPs and arginine (R) in TPs.

## Results

### HA-RAMPs display varying degrees of similarity to TPs

**Figure 1:**
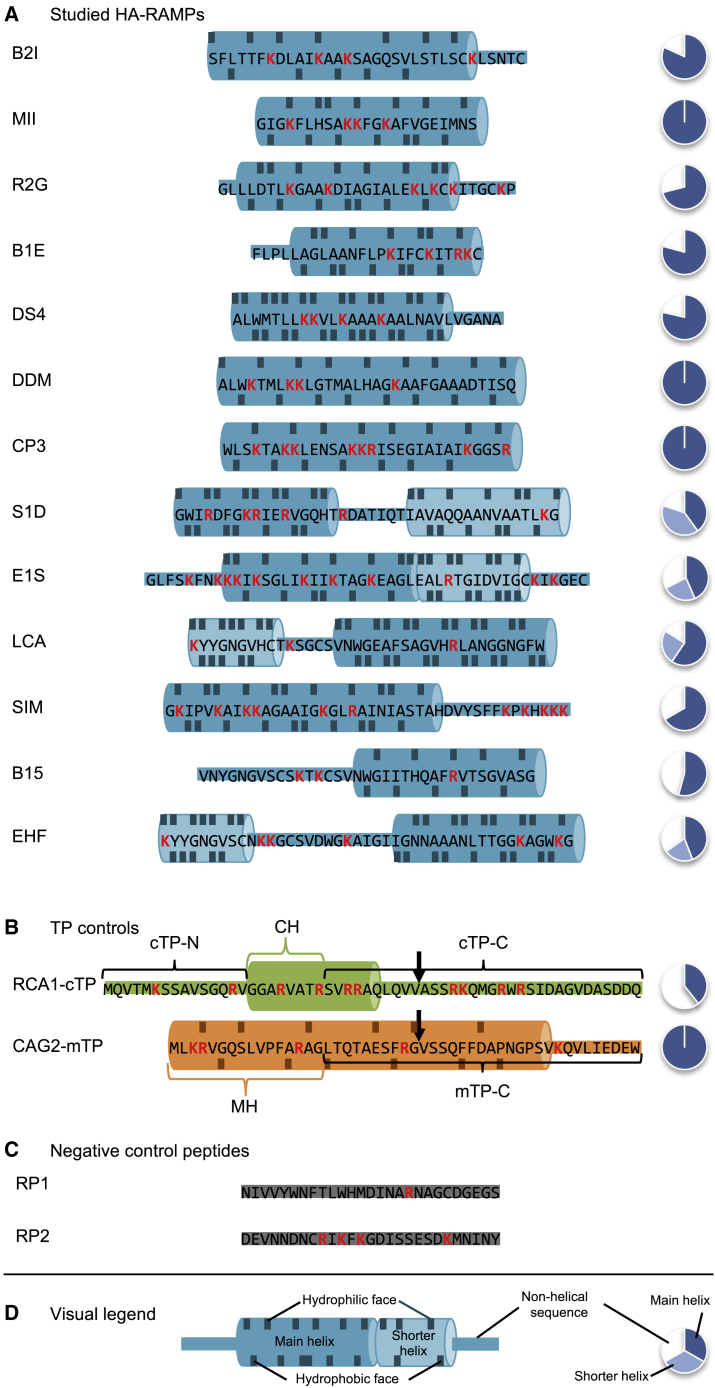
Peptide sequences under study. **(A–D)** Amino acid sequences are shown using the one-letter code. Positively charged residues are highlighted in red. The fraction of the sequence predicted to fold into amphipathic helices (Methods) is provided by a pie chart to the right of the sequence; for TPs in **(B)**, this was calculated up to the cleavage site indicated by a downward arrow. Predicted amphipathic helices are highlighted using a cylinder cartoon, with residues contributing to the hydrophilic/hydrophobic face indicated on the top/bottom. A visual legend is provided in **(D)**. No helix could be predicted within RCA1-cTP, and thus the indicated helix is taken from a published NMR structure obtained under membrane-mimetic conditions (Krimm et al., 1999). Note that the two helices of E1S are at an angle to each other and therefore cannot form a single continuous amphipathic helix. B2I, brevinin-2ISb; R2G, ranatuerin-2G; MII, magainin 2; B1E, brevinin-1E; DS4, dermaseptin S4; DDM, dermadistinctin-M; CP3, cecropin-P3; S1D, sarcotoxin-1D; E1S, esculentin-1SEA; LCA, leucocin-A; SIM, SI moricin; B15, bacillocin 1580; EHF, enterocin HF; TP, targeting peptide; cTP, chloroplast TP; mTP, mitochondrial TP; RCA1, Rubisco activase; CAG2, γ-carbonic anhydrase; cTP-N, cTP N-terminal element; CH, cTP helix; MH, mTP helix; cTP-C, cTP C-terminal element; mTP-C, mTP C-terminal element; RP, random peptide.

Figure 1 shows the major sequence features of the 13 HA-RAMPs used in the present study (Figure 1A) together with those of a typical cTP and a typical mTP (Figure 1B). On the right side, these peptides are represented according to their proportion of α-helical amphipathic structure. The HA-RAMPs brevinin-2ISb (B2I), magainin 2 (MII), ranatuerin-2G (R2G), dermaseptin S4 (DS4), dermadistinctin-M (DDM), brevinin-1E (B1E), cecropin-P3 (CP3), sarcotoxin-1D (S1D), esculentin-1SEA (E1S), leucocin A (LCA), SI moricin (SIM), bacillocin 1580 (B15), and enterocin HF (EHF) were chosen so that different AMP families with varying proximity to TPs would be represented (Supplemental Table 1) and, thus, a range of physico-chemical properties would be explored (Supplemental Figure 1). As negative controls, two peptides that lack predicted amphipathic helices were chosen from among randomly generated sequences (Figure 1C).

**Figure 2:**
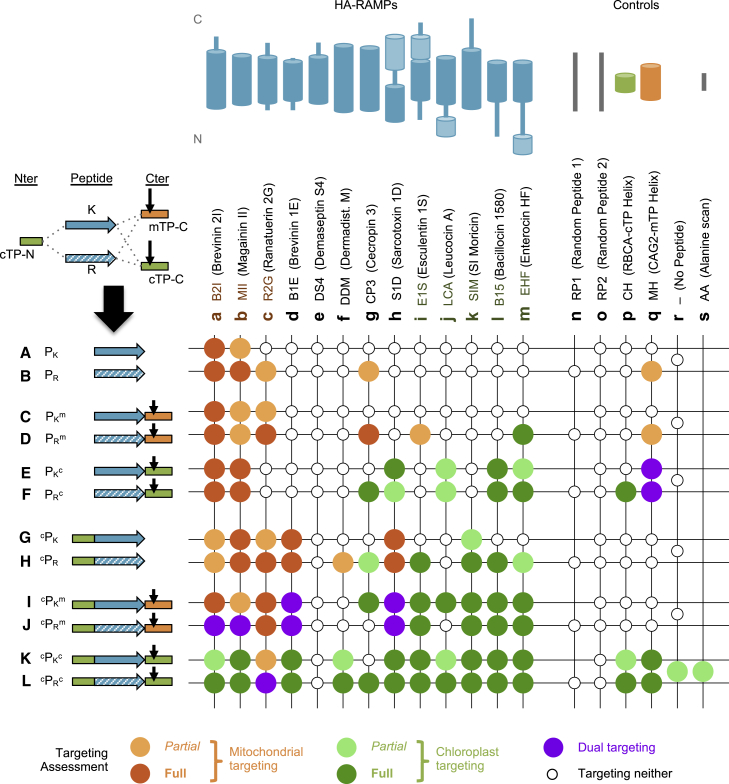
TP modifications enable HA-RAMP targeting. Chimeric constructs were generated by combining TP elements with HA-RAMPs (see Figure 1 for sequences; in the ^c^AA^c^ construct in column s, all residues of the helical element “CH” within RCA1-cTP are replaced by alanines). The overview graph shows in each column (a–s) one of the peptides, with a cartoon indicating the position of the predicted amphipathic helices within the sequence, and in each row (A–L) a combination of peptide, K/R modification, addition of cTP-N (the 15 N-terminal residues upstream of the helix in RCA1-cTP), and/or a C-terminal TP element (cTP-C or mTP-C, which include −10 residues upstream and +23 residues downstream of the cleavage site for RCA1-cTP and CAG2-mTP, respectively), indicated by a cartoon and the following shorthand: P, peptide; _K_, contains mostly Ks; _R_, contains mostly Rs; ^m^, mTP element; ^c^, cTP element (^c^P = cTP-N, P^c^ = cTP-C). In each case, an overview of observed targeting is provided by a color code. Images for all constructs are shown in Supplemental Figures 3–20. Note that the present results for B2I_K_^c^, MII_K_^c^, S1D_K_^c^, B15_K_^c^, and EHF_K_^c^ (row E, columns a, b, h, l, and m) confirmed our previous report on these strains (Garrido et al., 2020).

**Figure 3:**
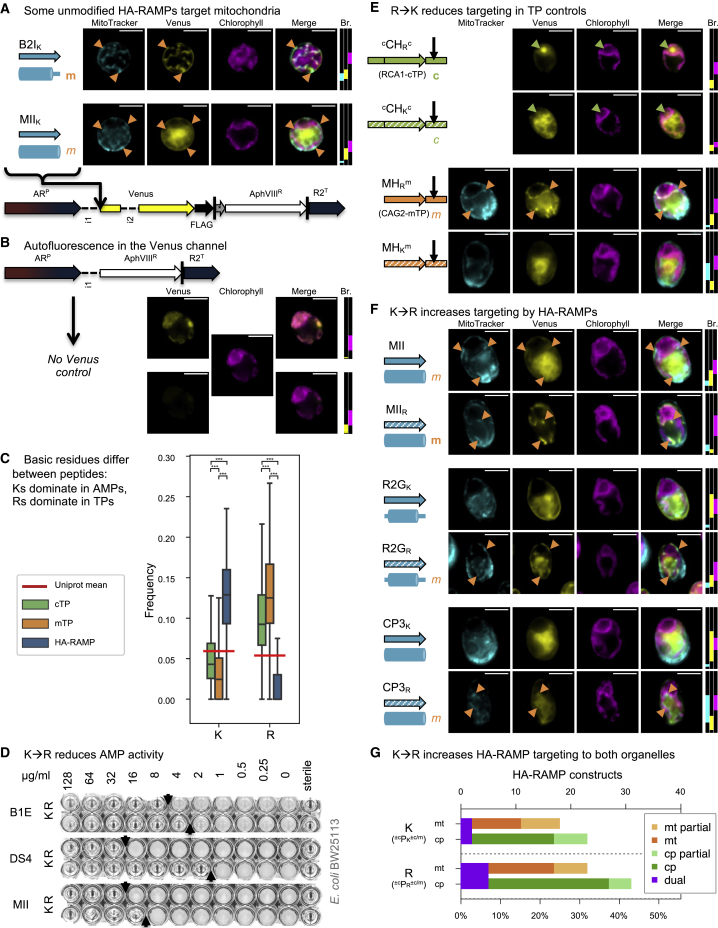
K is for killing, R is for targeting. **(A)** Indicated peptides were inserted upstream of a Venus fluorescent protein reporter in a bicistronic expression system for *Chlamydomonas* (Caspari, 2020). AR^P^, hybrid *HSP70a*-*RBCS2* promoter; i1, *RBCS2* intron 1; i2, *RBCS2* intron 2; FLAG, FLAG tag; |, stop codon; ∗, bicistronic bridge sequence tagcat; AphVIII^R^, paromomycin resistance gene; R2^T^, *RBCS2* terminator. Epifluorescence microscopy images of selected examples are shown. False-colored yellow fluorescence from the Venus channel reports on the subcellular localization of the fluorescent reporter. MitoTracker fluorescence, false-colored in cyan, indicates the position of mitochondria (although parts of the cell exterior are sometimes also stained), with salient features highlighted with orange arrows to indicate co-localization with the Venus channel. Chlorophyll autofluorescence, shown in magenta, indicates the location of the chloroplast. Scale bars, 5 μm. Refer to Figure 1 for sequences and Supplemental Figures 3 and 4 for biological replicates. Where a construct was interpreted as generating reporter localization in mitochondria or chloroplasts, this is indicated by an orange “m” or a green “c,” respectively, in bold for full targeting or in italics for partial targeting. Brightness (Br) was adjusted for clarity; fluorescence intensity values were restricted to the range shown for each channel by matching colored rectangles. Intensity scales to 0 at the bottom of the panel and to 65 535 at the top. **(B)** A “no Venus” control strain, expressing an empty vector, is shown with two different Venus channel Br settings to visualize chloroplast autofluorescence in the Venus channel. Autofluorescence intensity is typically below 2000, indicated by a black dotted line in Br rectangles. Therefore, if Venus channel Br is adjusted below 2000, then autofluorescence originating from the chloroplast may be misinterpreted as Venus located in the chloroplast. **(C)** Lysine (K) and arginine (R) frequencies for *Chlamydomonas* TPs and HA-RAMPs are shown as boxplots (center line, median; box limits, upper and lower quartiles; whiskers, min/max values within 1.5× interquartile range). To give a baseline for comparison, their average across the UNIPROT database is given as a red horizontal line. Statistically significant differences are indicated with asterisks (multiple Kruskal–Wallis tests followed by Dunn post hoc tests, ∗∗∗ *p* < 0.0001). See Supplemental Figure 21 for all amino acids. **(D)** Grayscale photos of antimicrobial activity growth assays in the presence of dilutions of three selected AMPs against *Escherichia coli* strain BW25113 show reduced activity when natively K-rich sequences (K, left columns) are altered by replacing all Ks with Rs (R, right columns). **(E and F)** Epifluorescence images of selected chimeric **(E)** TP controls (MH, mTP helical fragment; CH, cTP helical fragment) and **(F)** HA-RAMP constructs are shown as in **(A)**. Note that images shown for MII_K_ in **(A)** and **(F)** come from independent insertion lines of the same construct. See Supplemental Figures 4, 5, 9, 18, and 19 for biological replicates. **(G)** Data for targeting across all HA-RAMP constructs (i.e., including additional modifications) are shown, comparing K- and R-bearing peptides (cf. Figure 2, columns a–m).

**Figure 4:**
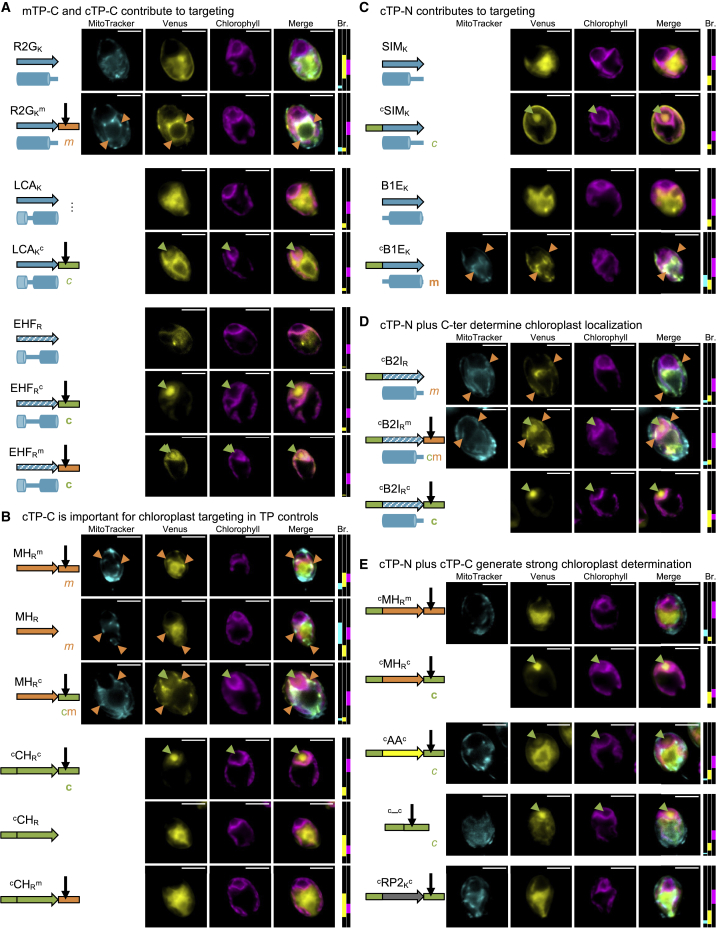
TP N- and C-termini enable chloroplast targeting by HA-RAMPs. **(A–E)** Exemplary epifluorescence images are shown as in Figure 3; same convention as in Figure 3 for Venus localization. Note that images shown for MH_R_^m^ and ^c^CH_R_^c^ in **(B)** come from different independent insertion lines of the same constructs as in Figure 3E. See Supplemental Figures 3–20 for biological replicates and further examples of the same trends.

To explore targeting, the 13 HA-RAMPs were systematically modified (Figure 2), notably by adding TP elements from the N- and/or C-terminal domains of *Chlamydomonas* Rubisco activase (RCA1) cTP or a C-terminal domain of similar length from mitochondrial γ-carbonic anhydrase 2 (CAG2) mTP (Figures 1B and 2). In a bid to keep peptidase recognition sites intact, C-terminal elements were designed to start 10 residues upstream of the cleavage site, even though this slightly truncates TP helices. Peptides carrying the 15-amino-acid cTP N-terminus (cTP-N) will be denoted ^c^P. Similarly, peptides with C-terminal elements from RCA1-cTP (cTP-C) or CAG2-mTP (mTP-C) will be denoted P^c^ or P^m^, respectively. Peptide variants are used to drive the subcellular localization of a Venus fluorescent reporter protein, which was assessed using fluorescence microscopy; in Figure 2, an executive overview of observed Venus localization is presented for all constructs. We previously validated fluorescence localization biochemically in a small number of strains by showing that the Venus-FLAG reporter is retained within isolated mitochondria or chloroplasts (Garrido et al., 2020). Here, we use automated image segmentation to validate our subcellular localization assessment (Supplemental Figure 2, Supplemental text). Localization was obtained using stable *Chlamydomonas* expression lines generated by introducing DNA sequences encoding peptides upstream of the Venus coding sequence in a bicistronic expression vector (Caspari, 2020) using Gibson assembly, with transformation cassettes integrated into the *Chlamydomonas* nuclear genome at random sites via electroporation. Micrographs for three biological replicates (i.e., independent insertion lines) per construct are displayed in Supplemental Figures 4–21. The reader will be asked to return to Figure 2 throughout, with Figures 3 and 4 providing selected micrographs as examples highlighting particular points of interest.

### Some unmodified HA-RAMPs generate mitochondrial targeting

In the absence of any modifications, B2I and MII were capable of organelle targeting visible in fluorescence microscopy (Figure 2, row A, and Supplemental Figures 3–15). When equipped with B2I, the fluorescent reporter Venus shows mitochondrial localization (Figure 3A and Supplemental Figure 3); the Venus signal is observed as a characteristic network pattern that co-localizes with MitoTracker fluorescence (cf. Supplemental Figures 2A and 4). By contrast, in the case of MII, only a fraction of the fluorescence signal in the Venus channel shows co-localization with the MitoTracker dye, signifying that targeting is only partial (Figure 3A and Supplemental Figure 5). Note that in these epifluorescence images, some autofluorescence emanating from the chloroplast is present in the Venus channel (Figure 3B). In all images, display brightness was adjusted so that Venus localization would be clearly visible, and where possible, this included removal of autofluorescence. Brightness settings were chosen as indicated next to each image for full transparency. In cases where low Venus accumulation necessitated brightness values low enough for autofluorescence to be visible in the Venus channel, a black dotted line is visible in the brightness display, indicating that some autofluorescence signal co-localizing with chlorophyll should be expected in the Venus channel independent of the genuine Venus localization.

### K/R content contributes to functional divergence between HA-RAMPs and TPs

Extant HA-RAMPs and organellar TPs display very few differences in their amino acid content (Supplemental text, Supplemental Figure 20). As expected, both are poor in acidic residues (D and E) but enriched in basic residues (K or R). However, their complement of basic residues is markedly different (Figure 3C); HA-RAMPs are rich in Ks, whereas TPs are rich in Rs.

To see whether these contrasting differences in K/R ratio contributed to the functional divergence between HA-RAMPs and TPs, we substituted all instances of K with R in HA-RAMPs and of R with K in TPs. In the rest of the text, the basic amino acid mostly present in a given peptide P is indicated by a subscript (P_R_ or P_K_). HA-RAMPs with a K→R transition showed reduced antimicrobial activity, as illustrated in Figure 3D by the increased minimum inhibitory concentrations for MII, DS4, or B1E.

We used RCA1 cTP (^c^CH_R_^c^) and CAG2 mTP (MH_R_^m^) as positive controls for chloroplast and mitochondrial targeting, respectively (Figure 3E and Supplemental Figures 18 and 19). Note that TP helical fragments, stripped of their N- and C-terminal domains, are denoted as MH for mTP and CH for cTP (as detailed in Figure 1B). When equipped with RCA1-cTP (^c^CH_R_^c^), Venus shows two chloroplast localization features: a diffuse signal that colocalizes with chlorophyll fluorescence, and a bright spot where there is a drop in chlorophyll fluorescence. Both of these features are genuine markers of chloroplast localization: in *Chlamydomonas*, the single cup-shaped chloroplast has a reliable morphology, and the dip in chlorophyll fluorescence at the base of the chloroplast is a well-established marker of the pyrenoid, a proteinaceous chloroplast sub-compartment that contains a large majority of Rubisco (Supplemental Figure 2B; Mackinder et al., 2016, 2017; Caspari et al., 2017; Caspari, 2022). Although small proteins like Venus can enter the pyrenoid, most thylakoid membranes are excluded, leading to the observed decrease in chlorophyll autofluorescence in this spot. A Venus channel signal emanating from the pyrenoid is thus a useful visual guide to true chloroplast localization of the fluorescent reporter (Caspari, 2022). Note that, because cTP-C contains a Rubisco-binding motif (Meyer et al., 2020), Venus accumulation in the Rubisco microcompartment, the pyrenoid, is particularly pronounced in constructs carrying this element. When natively R-rich RCA1-cTP and CAG2-mTP sequences were subjected to systematic R→K substitutions (^c^CH_K_^c^, MH_K_^m^), their ability to target was reduced or abolished (Figure 3E and Supplemental Figures 18 and 19). These experiments demonstrate the respective functional contributions of K and R residues to antimicrobial and organelle targeting activity.

We then systematically re-examined the organelle targeting ability of the set of 13 HA-RAMPs that had undergone K→R substitutions (Figure 2, row B, and Supplemental Figures 3–15). MII_R_ now shows much improved mitochondrial targeting (Figure 3F), with a large majority of Venus colocalizing with MitoTracker (note that the MitoTracker occasionally appears to stain the cell envelope in addition to the mitochondria; thus, not all of the MitoTracker signal colocalizes with Venus; cf. Supplemental Figure 2A). R2G_R_ as well as CP3_R_ show a gain of partial targeting, as evidenced by significant overlap of Venus and MitoTracker fluorescence (Figure 3F). Thus, a substitution of K for R increases targeting by HA-RAMP constructs. This effect is not exclusive to mitochondrial targeting (Figure 3G). Across constructs (i.e., including those containing additional modifications), the K→R switch enabled or improved mitochondrial targeting in 8 cases (Figure 2, rows = capital letters, columns = lower case letters; gain: ABcg, CDgi, GHf; improve: ABb, CDc, GHc) and chloroplast targeting in 15 cases, including 3 cases of dual targeting (Figure 2, gain: CDm, EFg, GHgilm, KLg; improve: EFm, GHk, KLafj; dual: IJab, KLc). Lost or decreased targeting was observed in only three cases (Figure 2, loss: IJgj, decrease: EFh). See Supplemental Figure 24 for illustrative examples.

### TP C-termini matter for targeting

Addition of mTP-C, the cleavage-site-containing C-terminal element of CAG2-mTP, enabled partial mitochondrial targeting in two constructs (R2G_K_ and E1S_R_) and improved mitochondrial targeting in two more constructs (R2G_R_ and CP3_R_), in addition to continued targeting by B2I and MII (Figure 2, rows C and D). As an indicative example, gain of targeting in R2G_K_ is shown in Figure 4A. The impact of adding cTP-C, the C-terminal element derived from RCA1-cTP, is even more important: cTP-C significantly enabled chloroplast targeting, which could be seen in nine HA-RAMP constructs involving CP3_R_, S1D, LCA, B15, and EHF (Figure 2, rows E and F). Note that the addition of cTP-C was also compatible with mitochondrial localization by B2I and MII (Figure 2, rows E and F). Gain of partial chloroplast targeting in LCA_K_ and full targeting in EHF_R_ are shown as examples in Figure 4A. The addition of mTP-C also enabled chloroplast localization by EHF_R_ (Figures 4A and 2, row D). Low Venus accumulation in EHF_R_ and EHF_R_^m^ means that the brightness needed to be set low enough that autofluorescence accounts for at least some of the signal that is colocalized with the chlorophyll channel (Figure 4A). EHF_R_ shows a Venus signal in the cytoplasm but not within the pyrenoid, which was interpreted as an absence of targeting. By contrast, the Venus signal emanating from within the pyrenoid in EHF_R_^m^ provides unambiguous evidence for chloroplast localization.

In TP controls, deletion of mTP-C reduces, but does not totally abolish, mitochondrial targeting of CAG2-mTP (MH_R_), whereas replacing mTP-C with cTP-C (MH_R_^c^) partially retargets the construct to the chloroplast (Figures 4B and 2, column q, rows B, D, and F). By contrast, deletion of cTP-C abolishes chloroplast targeting by RCA1-cTP (^c^CH_R_), as does replacing cTP-C with mTP-C (^c^CH_R_^m^) (Figures 4B and 2, column p, rows H, I, and L).

### cTP N-termini matter for chloroplast targeting

The sole addition of cTP-N generated at least partial chloroplast targeting in 6 HA-RAMP constructs, notably by SIM (Figure 4C) but also by CP3_R_, E1S_R_, B15_R_, and EHF_R_ (Figure 2, rows G and H). However, cTP-N also enabled mitochondrial targeting in three HA-RAMPs that had not previously shown mitochondrial localization: B1E (Figure 4C), DDM_R_, and S1D (Figure 2, rows G and H).

The importance of cTP-N as a chloroplast determinant becomes more evident when combined with a C-terminal element, as shown in Figure 4D using the example of ^c^B2I_R_. While ^c^B2I_R_ (partially) targets the mitochondria, addition of mTP-C (^c^B2I_R_^m^) results in dual targeting to the chloroplast in addition to the mitochondria, and addition of cTP-C (^c^B2I_R_^c^) results in targeting only to the chloroplast. Across HA-RAMP constructs, combining cTP-N with mTP-C (Figure 2, rows I and J) resulted in 6 cases of dual targeting (B2I_R_, MII_R_, B1E, and S1D) and 10 cases of chloroplast targeting (CP3_K_, E1S, LCA_K_, SIM, B15, and EHF). Combining cTP-N with cTP-C (Figure 2, rows K and L) generated at least partial chloroplast targeting in 22 of 26 HA-RAMP constructs (including 1 instance of dual targeting by R2G_R_); only DS4 failed to show any chloroplast targeting.

Finally, Figure 4E (also Figure 2, column q, rows J and L) shows that, while addition of cTP-N to the CAG2-mTP abolished mitochondrial targeting (^c^MH_R_^m^), the cTP-N/cTP-C combination (^c^MH_R_^c^) retargets to the chloroplast (see Figure 4B for controls without cTP-N). Replacing the native amphipathic helix of RCA1-cTP between cTP-N and cTP-C with a poly-alanine peptide of equal length (^c^AA^c^) or fusing cTP-N and cTP-C directly with no intervening peptide (^c^-^c^) lead to partial chloroplast targeting (Figures 4E and 2, rows K and L, columns r and s). The two latter experiments demonstrate that there are enough determinants for recognition of the chloroplast translocon dispersed between the N-terminus and C-terminus of a cTP to target Venus to the chloroplast, albeit with a lower efficiency than when an amphipathic helix is present in between. That the nature of the intervening peptide matters can be further seen in constructs that fail to target the chloroplast in the presence of cTP-N and cTP-C, such as random peptide 2 (RP2) (Figure 4E) and also RP1, CP3_K_, DS4, and R2G_K_ (Figure 2, rows K and L).

### HA-RAMPs dominate targeting specificity

Considering Figure 2 by columns reveals that organelle specificity is, to a large extent, determined by HA-RAMPs. Only 3 HA-RAMPs (B2I, R2G, and MII; referred to hereafter as the mt set) account for more than 70% of all constructs in which mitochondrial targeting is seen. Similarly, 5 HA-RAMPs (E1S, LCA, SIM, B15, and EHF; referred to as the cp set) account for the majority (∼57%) of all chloroplast targeting and for two thirds when excluding the cTP-N/cTP-C combination that generates chloroplast targeting across most HA-RAMPs. In some instances, a set of peptide modifications may switch targeting from the mitochondria to the chloroplast, but the major effect of modifications—i.e. exchanging K→R (Figure 3 and Supplemental Figure 24) or adding TP elements (Figure 4)—is to enhance the targeting ability to an organelle determined by the HA-RAMP primary sequence properties (Figure 2).

### Probing cleavage of HA-RAMP-driven reporter constructs by immunoblotting

**Figure 5:**
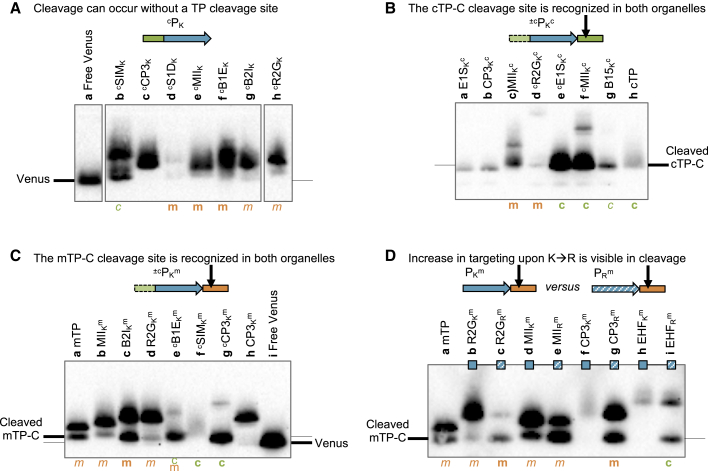
Import is associated with maturation of the preprotein. **(A–D)** Western blots used an α-FLAG antibody on selected constructs, as indicated above the blots. Where a construct was interpreted as generating reporter localization by fluorescence microscopy (cf. Figure 2 and Supplemental Figure 3–20) in mitochondria or chloroplast, this is indicated by an orange “m” or a green “c,” respectively, in bold for full targeting or in italics for partial targeting. In **(A)**, some lanes were spliced for clarity; the uncropped blot is provided in Supplemental Figure 28. The migration of Venus without any presequence and Venus with additional amino acids at the N-terminus left over after cleavage of cTP-C and mTP-C is indicated for reference based on free Venus and cTP/mTP controls, respectively. The cTP control is RCA1-cTP (construct ^c^CH_R_^c^), and the mTP control is CAG2-mTP (construct MH_R_^m^).

To characterize the maturation of HA-RAMP-targeted proteins upon organellar import, we performed immunoblotting experiments using whole-cell extracts probed with a FLAG antibody targeting the Venus-FLAG reporter (Figure 5). Indicative examples were selected for clarity; a more comprehensive overview is provided in Supplemental Figure 28.

In the absence of a dedicated cleavage site (Figure 5A), we found that some processing did occur, but preproteins are also maintained, as evidenced by the presence of two bands in a given lane. The upper band corresponds to unprocessed Venus, which is a fusion of the reporter with the HA-RAMP construct and migrates at varying positions depending on the length of the presequence. The lower band corresponds to the processed form migrating closer to the “free” Venus position (Figure 5A, lane a), depending on the exact site of cleavage. In HA-RAMP constructs, the majority of Venus remained in the unprocessed top band for constructs targeting the mitochondria (Figure 5A, lanes d–h). Nonetheless, the presence of a faint processed form as lower bands just above free Venus shows that some processing occurs. Such processing is suggestive of import but is not conclusive because proteolysis can occur unlinked to import. The proportion of processed versus unprocessed Venus preprotein is much higher in partially chloroplast-targeted ^c^SIM_K_ (Figure 5A, lane b), hinting at more efficient degradation of the unprocessed form in the chloroplast.

Addition of a cleavage site improves processing across organelles. In the presence of cTP-C, which contains the RCA1-cTP cleavage site, HA-RAMP constructs that show evidence of organellar targeting appear to be processed at a site corresponding to the cTP control (Figure 5B, lane h), independent of whether the mitochondria or the chloroplast are targeted (Figure 5B, lanes c–g). Constructs that do not target (Figure 5B, lanes a and b; see also Supplemental Figure 28C, lanes a, e, and i) also appear to be processed, but at a site different from the one used in organelles, suggesting that cTP-C may be recognized by cytoplasmic peptidases.

Figure 5C shows constructs equipped with mTP-C, which contains the CAG2-mTP cleavage site. Here, the mTP control (Figure 5C, lane a) shows two bands, consistent with the partial targeting observed by microscopy (cf. Figures 3E and 4B). The top band thus likely corresponds to the preprotein and the bottom band to the mature form within the mitochondria. HA-RAMP constructs equipped with mTP-C that target either mitochondria or the chloroplast (Figure 5C, lanes b–g) show lower bands that migrate at or near the cleaved mTP control. These results indicate that mTP and cTP cleavage sites are recognized in both organelles.

A switch from K to R, which improved targeting, also increased the amount of processed form relative to that of the unprocessed form in all cases, whether because of a gain of targeting (Figure 5D, lanes f–i) or an increase in efficiency (Figure 5D, lanes b–e), suggesting that cleavage can serve as a proxy for targeting. Indeed, constructs that show targeting, as judged from microscopy, also show evidence of cleavage in immunoblots; second bands are present for constructs lacking cleavage sites (Supplemental Figure 28A and 28D), and cleavage-site-containing constructs migrate at the size expected for processed cTP-C (Supplemental Figure 28C and 28F) or mTP-C (Supplemental Figure 28B and 28E). We note that faint processed bands can be seen for several additional constructs (Supplemental Figure 28), notably for unmodified HA-RAMPs (Supplemental Figure 28A), suggesting that partial targeting may occur in these cases, backed up by high image quantification values; e.g., for CP3_K_ and R2G_K_ (Supplemental Figures 2E, 2F, 5A, and 9A). Such very partial targeting is, however, below the detection limit of our targeting assessment based on fluorescence microscopy.

### Chloroplast targeting involves longer, less helical peptides

**Figure 6:**
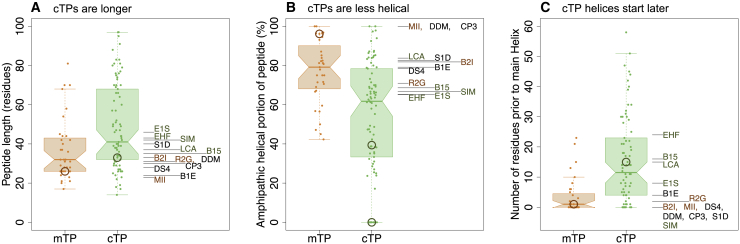
Chloroplast- and mitochondrion-targeting HA-RAMPs match cTPs and mTPs, respectively. **(A–C)** For salient properties, *Chlamydomonas* mTPs and cTPs are compared with our 13 HA-RAMPs. TP distributions are shown as boxplots (center line, median; box limits, upper and lower quartiles; whiskers, min/max values within 1.5× interquartile range), and colored points represent individual peptides. The position of CAG2-mTP and RCA1-cTP is circled in each graph. The non-zero value for RCA1-cTP in **(B)** and the single circle in **(C)** report on the amphipathic helix established by an NMR study (Krimm et al., 1999) because no helix could be predicted by our approach. HA-RAMPs are color coded by preferred targeting: orange, mt set; green, cp set (cf. Figure 2). **(A)** cTPs are significantly longer than mTPs (*p* = 0.0017), and cp-set are longer than mt-set HA-RAMPs (*p* = 0.0184). **(B)** Helices make up a significantly smaller fraction of cTPs than mTPs (*p* < 0.0001), and HA-RAMPs show a similar trend (0.1122). **(C)** cTPs contain significantly longer sequence stretches upstream of the longest predicted helix than mTPs (*p* < 0.0001), as do cp-set compared with mt-set HA-RAMPs (*p* = 0.0205). Reported *p* values were obtained through two-way *t*-tests for TPs and one-way *t*-tests for HA-RAMPs.

To understand what differentiates mt-set from cp-set HA-RAMPs, we compared the sequence characteristics of our 13 HA-RAMPs with those of well-characterized *Chlamydomonas* TPs (Figure 6). In *Chlamydomonas*, cTPs are, on average, 49 residues in length (Figure 6A) and significantly longer than mTPs (*t*-test, *p* = 0.0017), which are, on average, 37 residues in length. The difference is even greater than shown here, given that many cTPs require a contribution from post-cleavage site residues for successful targeting (Bionda et al., 2010; Caspari, 2022). Consistent with this, cp-set HA-RAMPs (green, Figure 6A) are longer than mt-set HA-RAMPs (orange, Figure 6A; *p* = 0.0184) and require further elongation by addition of TP elements before targeting can be observed (Figure 2).

Most mTPs fold into an amphipathic helix for ∼80% of the sequence on average (Figure 6B), starting directly from their N-terminus (Figure 6C). The fraction dedicated to amphipathic helix formation in cTPs is significantly lower (Figure 6B; *p* < 0.0001), with a longer stretch of non-helical sequence at the N-terminus (Figure 6B; *p* < 0.0001). In line with this contrast, 4 out of 5 of the cp-set HA-RAMPs have longer sequence stretches upstream of the main helix than the mt-set HA-RAMPs (Figures 1 and 6C; Franzén et al., 1990; Supplemental text, Supplemental Figures 22 and 23). Also note that cTPs are predicted to be more prone to protein interaction than mTPs (Supplemental text, Supplemental Figures 26 and 27).

## Discussion

### How to make TPs from HA-RAMPs

**Figure 7:**
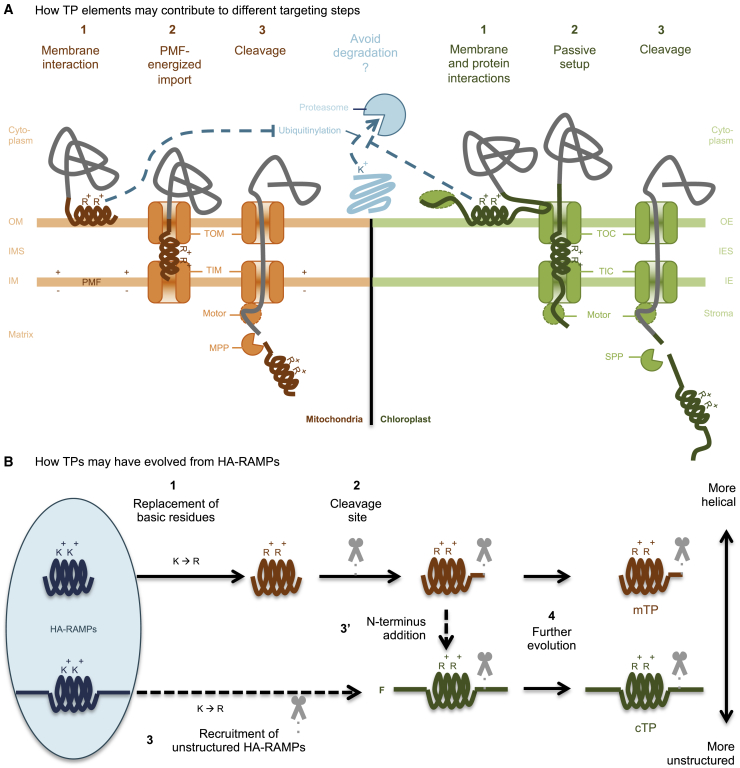
Proposed functioning and evolution of TPs. OM, outer membrane; IMS, inter-membrane space; IM, inner membrane; OE, outer envelope; IES, inter-envelope space; IE, inner envelope; PMF, proton motive force; TOM, translocon of the outer mitochondrial membrane; TIM, translocon of the inner mitochondrial membrane; TOC, translocon of the outer chloroplast envelope; TIC, translocon of the inner chloroplast envelope; MPP, matrix processing peptidase; SPP, stromal processing peptidase; F, phenylalanine; mTP, mitochondrial targeting peptide; cTP, chloroplast transit peptide. **(A)** A number of roles for TP elements during protein import are suggested. Because ubiquitination targets K residues, a preference for R in TPs may increase preprotein stability. R may also play a role in lipid interactions. (1) Membrane interactions may play a role in enabling differential targeting because of organelle-specific lipid preferences of TP helices. Protein interactions by cTP N- and C-terminal elements (e.g., with cytosolic factors or TOC components [subunits are not shown for simplicity]) may also play a role in specific targeting. (2) Import of positively charged mTPs across the inner membrane is energized by the proton motive force. By contrast, a passive setup is required for cTPs to allow N-termini to reach into the stroma and contact the motor complex, likely contributing to increased length and relatively unstructured N- and C-termini of cTPs. (3) Sequence elements contributing to targeting may be present downstream of cleavage sites in cTPs. **(B)** If TPs evolved from AMPs, then (1) HA-RAMPs would have first changed K to R and (2) acquired a cleavage site to become mTPs. To generate cTPs, either (3) more unstructured HA-RAMPs were recruited directly to become cTPs by undergoing a K-to-R shift and acquiring a cleavage site separately, or (3′) mTPs acquired an N-terminal non-helical domain. Early cTP N-termini likely contained a starting F (Wunder et al., 2007). (4) Further evolution would have reinforced the differences between cTPs and mTPs to limit mis-targeting.

To gain insight into peptide features that govern targeting, we used a related but separate class of peptides, HA-RAMPs, as a chassis and stacked modifications to generate targeting into mitochondria or chloroplasts or, in some cases, into both (Figure 7A).

### Rs target better than Ks

We showed that a K-to-R-switch increases targeting efficacy to both organelles across many constructs. R is more common than K in cTPs across green algae, vascular plants, red algae, glaucophytes, and many secondary plastids (Patron and Waller, 2007), but the functional significance for targeting efficacy had not been recognized previously. Our observation is in line with a previous report that a R-to-K switch at the N-terminus of an *Arabidopsis* mTP abolished targeting (Lee et al., 2019). The underlying mechanism warrants further research. Contributing factors may be differences in bulkiness and pKa (Li et al., 2013, 2017) or in *trans*-acting factors that regulate targeting. For example, R features in consensus sequences of the cleavage sites (Tardif et al., 2012; Calvo et al., 2017), and recognition of R by processing peptidases may thus potentially account for increased chloroplast localization among cTP-N-bearing constructs (Figure 2, rows G and H). Because K is used for ubiquitination (Mattiroli and Sixma, 2014), a preference for R over K in TPs may help to protect preproteins from degradation while they transit the cytosol (Figure 7A). We note that the 20S proteasome is already present in archaea, with effectors specifically targeting K, as in the eukaryotic cytosol (Maupin-Furlow et al., 2006; Maupin-Furlow, 2013), which means that K residues were likely targets for protein degradation when primary endosymbiosis led to formation of the proto-mitochondrion.

### Unstructured sequences contribute to chloroplast targeting

Differences between cTP and mTP N-termini have been recognized previously to contribute to differential targeting (Bhushan et al., 2006), but the sequence features underlying this differentiation have been a matter of debate. In vascular plants, the presence of an N-terminal multi-R motif has been shown to prevent chloroplast import, leading to a proposal that this feature was solely responsible for differential targeting (Lee et al., 2019, 2020). A different research effort focused on the presence of Hsp70 binding sites within cTP N-termini as crucial for enabling import (Chotewutmontri et al., 2012, 2017; Chotewutmontri and Bruce, 2015). Here, we found that multiple Rs are not uncommon in *Chlamydomonas* cTP N-termini (Supplemental Figure 23) and that Hsp70 binding sites are equally present in mTP N-termini (Supplemental Figure 26). We found that HA-RAMPs with intrinsically unstructured N-termini were able to support chloroplast targeting even in the absence of a cTP-N addition. This finding makes it extremely unlikely that differential import relies on specific peptide–receptor interactions mediated by co-evolved sequence motifs. Instead, we argue that the feature that differentiates cTPs from mTPs is the presence of an unstructured sequence upstream of the amphipathic helix in cTP N-termini. This view re-interprets the tripartite structure of cTPs (von Heijne et al., 1989), which is conserved all the way from glaucocystophyte algae to vascular plants (Köhler et al., 2015), by describing cTPs as composed of a central amphipathic helix flanked by N- and C-terminal unstructured sequence elements. This central helix has a weaker signal than the helix in mTPs (von Heijne and Nishikawa, 1991) and may only form upon membrane contact (Bruce, 2000; Garrido et al., 2020).

We also found that chloroplast targeting was further improved by unstructured C-termini. That TP C-termini are important to enable import has been noted previously, with cTP and mTP C-termini thought to be functionally interchangeable (Lee et al., 2019). Here, we found that the nature of the C-terminus does matter: the more unstructured cTP-C enabled chloroplast targeting more often and more effectively than mTP-C, which contains a long predicted amphipathic helix. The need to contain unstructured sequences (von Heijne and Nishikawa, 1991) as well as the increased length of cTPs, even extending beyond the cleavage site in many cases (Bionda et al., 2010; Caspari, 2022), could be mechanistically related to the import system (Figure 7A). Whereas mitochondrial import makes use of the proton gradient to power uptake of positively charged presequences (Martin et al., 1991; Garg and Gould, 2016), energized chloroplast import requires the cTP to stretch across the translocon of the outer chloroplast envelope (TOC) and the translocon of the inner chloroplast envelope and contact the translocation motor (Chotewutmontri et al., 2017; Nakai, 2018; Richardson et al., 2018). Structured sequence elements can thus impede import, including the helix at the Venus N-terminus (Rekas et al., 2002). Consistent with this view, HA-RAMPs with unstructured C-termini, such as SIM, generated chloroplast targeting in the absence of an added TP-C.

Unstructured elements also likely provide protein-protein interaction motifs. For instance, TOC interaction has been attributed to semi-conserved “FGLK” motifs (Pilon et al., 1995; Lee et al., 2009; Chotewutmontri et al., 2012; Holbrook et al., 2016), although the requirement for F appears to be relaxed in *Chlamydomonas* (Supplemental Figure 26E; Razzak et al., 2017). The presence of GLK sites with high predicted interactivity in cTP-N and cTP-C should contribute to the high chloroplast targeting potential of constructs equipped with both elements (Supplemental Figure 26).

### Amphipathic helices contribute to differential targeting

Although N- and C-terminal elements aid in specificity determination, particularly for chloroplast import, their influence fails to explain why some HA-RAMPs target preferentially the mitochondria or exclusively the chloroplast. This observation suggests that targeting specificity is also determined by some sequence properties of the amphipathic helical elements, which, in the case of HA-RAMPs, serve to mediate insertion into specific target membranes. It is noteworthy that individual mTPs and cTPs also interact with membrane bilayers (von Heijne et al., 1989; Bruce, 1998). For instance, the Rubisco small subunit cTP interacts with chloroplast-mimetic membranes only in the presence of the chloroplast-specific galactolipids (Pinnaduwage and Bruce, 1996). Taken together, these observations suggest that amphipathic helices may interact specifically with the membranes of the targeted organelle (Patron and Waller, 2007; Lazzaro et al., 2020). A direct interaction with the membrane bilayer, before interaction with proteins of the translocons, would provide a basic mechanism for a first step in differential organelle targeting (Figure 7A). It could explain how TPs can be functionally specific and diverse in sequence.

### A possible series of events for the evolution of TPs

A small subset of randomly chosen sequences is able to inefficiently deliver proteins into extant mitochondria, relying on amphipathic helices (Baker and Schatz, 1987; Lemire et al., 1989). Thus, the existing protein import machinery recognizes amphipathic helical peptides. However, an origin of TPs from random sequences does not explain how protein import into organelles would have spontaneously occurred in the absence of the extant translocons.

Here, our use of AMPs that harbor an amphipathic helix to further understand organelle targeting specificity was inspired by the hypothesis that HA-RAMPs may have given rise to TPs during endosymbiotic organellogenesis because of an “import and destroy” mechanism from HA-RAMP-resistant bacteria (Wollman, 2016; Caspari and Lafontaine, 2021). In this context, combining the organelle-targeting behavior of all constructs that convert antimicrobial peptides into TPs can be translated into a temporal evolutionary scenario (Figure 7B) that adds to previously proposed models (Lemire et al., 1989; Garg and Gould, 2016; Lee and Hwang, 2021). First, we found that several unaltered HA-RAMPs can deliver cargo, suggesting that the evolution of mTPs from HA-RAMPs would have been straightforward. However, K→R was found to reduce toxicity and increase targeting, a dual effect that would have produced a strong selection pressure favoring this exchange as an early step. Because mitochondria appear to tolerate the presence of preproteins containing unaltered HA-RAMPs, the addition of cleavage sites that allow presequences to be degraded separately from the cargo protein (Kmiec et al., 2014) would have come as a second step.

That chloroplast targeting requires additional discriminating elements is consistent with cTPs evolving in a cell that already had mTPs. There are two possible scenarios for the origin of cTPs. First, cTPs might have evolved directly from HA-RAMPs that already contained unstructured sequence elements (Figure 7B), as seen for our cp-set HA-RAMPs. Second, as suggested earlier (Lee and Hwang, 2021), cTPs might have co-opted existing mTPs. According to the present scenario, these mTPs already contained R and a cleavage site but would still have been recognized by cyanobacterial HA-RAMP importers. In this case, the key innovation that generated cTPs may simply have been the addition of unstructured, possibly TOC-interacting elements to mTPs, which we showed here to be sufficient to retarget the mt-set HA-RAMPs equipped with TP C-termini at least partially to the chloroplast. The cTP N-terminus likely originally started with an F, given that, in glaucophytes and rhodophytes (and many derived secondary plastids), a conserved N-terminal F plays a role in chloroplast import (Patron and Waller, 2007; Wunder et al., 2007; Köhler et al., 2015). This observation led to the idea that cTPs may have originated from a re-use of a C-terminal F-based motif involved in secretion via OMP85 beta-barrel proteins (Robert et al., 2006; Knopp et al., 2020) in (cyano)bacteria, from which TOC75 evolved. To this end, periplasmic polypeptide-transport-associated (POTRA) domains responsible for substrate recognition were proposed to have flipped orientation and now point into the host cytoplasm (Bullmann et al., 2010; Sommer et al., 2011). However, several subsequent studies of plant TOC75 have consistently found these domains to be in the inter-membrane space, not the cytoplasm (Chen et al., 2016; Paila et al., 2016; Gross et al., 2020). Without this cytoplasmic receptor, there is no mechanism for how an N-terminal F could have acted as an import-enabling proto-cTP (Knopp et al., 2020).

Note that our proposed scenario makes no prediction about whether host/proto-organelle interactions were mutualistic or antagonistic. Either way, AMPs are likely to have been part of the suite of tools used in these host/proto-symbiont interactions. In particular, the role of AMPs in mutualistic symbioses (Mergaert, 2018) includes one of the best-documented cases for defensive AMP import into bacteria (Guefrachi et al., 2015).

### Concluding remarks

Investigating the steps required to generate TPs from HA-RAMPs has allowed us to uncover a number of novel mechanistic insights. Notably, we discovered a role for Rs in targeting efficacy and delineated the contributions of N- and C-terminal elements in targeting specificity. Our work also suggests that, whether due to common descent or convergence, the similarities between TPs and HA-RAMPs point to TPs interacting with membrane lipids as an early targeting step. As any evolutionary scenario, our plausible pathway from HA-RAMPs to TPs must be considered as a working hypothesis that will need to be assessed further by a series of bioinformatics and laboratory-controlled evolutionary experiments. A better understanding of peptide–lipid interactions and the phylogeny of import machinery components should shed new light on the evolution and functioning of organelle TPs.

## Methods

### Construct generation

Venus expression constructs were designed in SnapGene (v.4.3.11) and generated by integrating PCR-amplified (Q5 Hot Start High Fidelity, M0515, New England Biolabs) DNA fragments into plasmid pODC53 (Caspari, 2020) upstream of Venus using Gibson assembly (NEBuilder HiFi DNA assembly, E5520S, New England Biolabs). *Chlamydomonas* TP sequences were amplified from genomic DNA extracted from strain T222+ (CC-5101). Templates for codon-optimized HA-RAMP, RP, and R→K modified TP sequences were obtained by gene synthesis (Eurofins Genomics). Correct assembly was verified by sequencing (Eurofins Genomics). Linear transformation cassettes were generated through restriction digestion with *Eco*RV (New England Biolabs).

### Transformation and fluorescence screen conditions

Constructs were transformed into wild-type strain T222+ (CC-5101) using a protocol described previously (Onishi and Pringle, 2016), except using 4 μl of DNA at 2 μg/μl. Transformants (≥24 per construct) selected for paromomycin resistance were grown in 200 μl Tris-Acetate-Phosphate-Medium (TAP) in 96-well plates under 50 μmol photons m^−2^ s^−2^ for 3–5 days and then screened for Venus expression in a fluorescence plate reader (CLARIOstar, BMG Labtech) as described previously (Caspari, 2020).

### Microscopy

Cells were grown in 96-well plates as described previously (Garrido et al., 2020). Strains with suspected mitochondrial targeting were treated with 0.1 μM MitoTracker Red CMXRos (Thermo Fisher Scientific) in growth medium for 30 min in the dark and washed with TAP prior to taking images. Epifluorescence microscopy was performed with cells added to 200 μl of either TAP or SEM (250 mM sucrose and 1 mM EDTA in 10 mM 3-(*N*-morpholino)propanesulfonic acid g, in poly-l-lysine (Sigma-Aldrich)-coated 8-well µ-slides (Ibidi) using the following setup: microscope, Axio Observer.Z1 inverted microscope (Zeiss); objective, α Plan-Apochromat 100×/1.46 oil DIC M27 (Zeiss); oil, Immersol 518 F (Zeiss); camera, ORCA-Flash4.0 digital camera (Hamamatsu); LEDs, 470 nm (chlorophyll), 505 nm (Venus), and white light (MitoTracker, filtered to >535 nm using Zeiss beam splitter 423052-0104-000) in the Colibri.2 LED system (Zeiss); filter cubes, filter 46HE YFP shift free (Zeiss, 520–550-nm emission) for Venus, custom-made filter set (559/34 BrightLine HC, Beamsplitter T 585 LP, 607/36 BrightLine HC; AHF Analysentechnik) for MitoTracker, and filter set 50 (Zeiss, 665–715-nm emission) for chlorophyll. Images were adjusted in Fiji (Schindelin et al., 2012) (ImageJ version 2.0.0) as described previously (Garrido et al., 2020), and final figures were assembled in PowerPoint (Microsoft PowerPoint for Mac 2011; version 14.6.3).

### Automated image analysis

A custom ImageJ Macro was written to enable automated image segmentation of epifluorescence micrographs in Fiji. In brief, in a given micrograph, fluorescence intensities were normalized, and individual cells were detected using marker-controlled watershed from the MorphoLibJ library (Legland et al., 2016). For each cell, auto-thresholding the chlorophyll channel using the Huang method was used to generate a chlorophyll mask. Separately, the chlorophyll channel was subjected to a series of morphological filters and Gaussian blurring followed by auto-thresholding to finally detect round holes between 0.1 and 5 μm within the ensuing binary image to be saved as a pyrenoid mask. In MitoTracker images, the MitoTracker channel was subjected to morphological filtering followed by auto-thresholding with Otsu to generate a mitochondrial mask. Venus channel intensities were recorded for each compartment. For each cell, rotated images containing binary masks as extra channels were saved. Data were compiled, analyzed, and plotted in R v.3.6.1 (https://www.r-project.org/) using RStudio 2022.07.2+576 (https://www.rstudio.com/).

### Western blots

Cells were grown in liquid culture (10 ml TAP, 30 μmol photons m^−2^ s^−1^, 160 rpm) until late mid-log phase. Two-milliliter aliquots were resuspended in 30 μl storage buffer (1× Roche cOmplete Mini proteinase inhibitor cocktail, 10 mM NaF, 0.2 M dithiothreitol, 0.2 M NaCO_3_) and stored at −20°C. Twenty microliters of boiling buffer (1× Roche cOmplete Mini proteinase inhibitor cocktail, 10 mM NaF, 50 g/l SDS, 200 g/l sucrose) was added, and then aliquots were boiled (50 s). Cell debris was removed (tabletop centrifuge, maximum speed, 15 min, 4°C). Chlorophyll content was estimated spectrophotometrically: 1 μg chlorophyll μl^−1^ = 0.11 × (optical density at 680 nm − optical density at 770 nm) for 5 μl diluted in 1 ml water. Samples (10 μg chlorophyll, equal volumes) were run (overnight, room temperature, 18 A) on large gels (35 × 27 cm; resolving gel: 12% acrylamide, 0.32% bisacrylamide, 0.25 μl/ml tetramethyl ethylenediamine, 250 mg/l ammonium persulfate, 375 mM Tris–HCl [pH 8.8]; stacking gel: 5% acrylamide, 0.133% bisacrylamide, 0.666 μl/ml tetramethyl ethylenediamine, 666 mg/l ammonium persulfate, 125 mM Tris–HCl [pH 6.8]). Proteins (<25 kDa, >75 kDa) were transferred (0.1 μm nitrocellulose membranes, 1 h, 0.8 A cm^−2^) as follows: cathode – 5 filter papers (FPs; 3 mm, Whatman) soaked in transfer buffer 1 (40 mM aminocaproic acid, 20% isopropanol, 25 mM Tris–HCl [pH 9.4]) – gel – membrane – 2 FPs soaked in transfer buffer 2 (20% isopropanol, 25 mM Tris–HCl [pH 10.4]) – 3 FPs soaked in transfer buffer 3 (20% isopropanol, 300 mM Tris–HCl [pH 10.4]) – anode. Membranes were fixed (Ponceau red), incubated (1 h, room temperature) in block (30 g/l skimmed milk powder, 0.1% Tween-20, 1× PBS: 140 mM NaCl, 30 mM KCl, 100 mM Na_2_HPO_4_, 15 mM KH_2_PO_4_), immunolabeled (overnight, 4°C) using α-FLAG primary antibody (Sigma-Aldrich F1804, diluted 1:10 000 in block), washed (0.1% Tween-20, 1× PBS), treated (1 h, room temperature) with horseradish peroxidase-conjugated α-mouse secondary antibody (diluted 1:10 000 in block), washed, and revealed (enhanced chemiluminescence [ECL]; ChemiDoc, Bio-Rad). Blots were processed using ImageLab (version 6.0.0 build 26, Bio-Rad), and final figures were assembled in PowerPoint (Microsoft).

### Antimicrobial activity assay

Minimum inhibitory concentration assays were performed as described previously (Garrido et al., 2020).

### Sequence dataset

TPs with experimentally confirmed cleavage sites were obtained from proteomics studies: *C. reinhardtii* cTP (Ge et al., 2014, Rowland et al., 2015, Terashima et al., 2011) and mTP (Tardif et al., 2012) and *Arabidopsis thaliana* cTP (Ge et al., 2014; Rowland et al., 2015) and mTP (Huang et al., 2009). For each peptide, we obtained the full-length protein sequence from NCBI and UniProt. Cytoplasmic control sequences were generated by taking N-terminal sequence stretches of random length (matching the distribution of peptide lengths observed in our *Chlamydomonas* TP dataset) from a random subset of *Chlamydomonas* proteins with validated cytoplasmic locations in UniProt. For principal-component analyses and calculation of amino acid frequencies, the same HA-RAMP, signal peptide, and non-*Chlamydomonas* TP sequences were used as before (Garrido et al., 2020).

### Amphipathic helix prediction

Amphipathic α helices were predicted as described previously (Garrido et al., 2020), following the principle of the HeliQuest algorithm (Gautier et al., 2008). In brief, this approach aims to establish the longest sequence stretch that contains identifiable hydrophilic and hydrophobic faces. The algorithm is iterated so that multiple non-overlapping helices can be found within a given peptide (Figure 1). Consequently, helix fractions are calculated as the number of residues within all predicted helices divided by the total number of residues in the peptide; to evaluate the number of upstream residues, only the longest helix was considered (Figure 6 and Supplemental Figure 25).

### Auto-cross covariance (ACC) terms

To evaluate the physicochemical properties of our peptides, we used the approach we described previously (Garrido et al., 2020). In brief, each amino acid is described in terms of 3 “Z-scale” values (Hellberg et al., 1987) that can be interpreted as representing a residue’s hydrophobic, steric, and electronic properties. ACCs between nearby residues are calculated up to a distance of 4 amino acids, generating a quantitative representation of a given peptide in terms of 36 ACC terms. Euclidian distances between HA-RAMP ACC term vectors and the barycenter of *Chlamydomonas* TPs were used as a measure of similarity (Supplemental Table 1).

### Visualization

We performed principal-component analysis to visualize the relationships among peptides as described by their 36 ACC terms (Supplemental Figures 1 and 22) or by their 5 salient TP properties (Supplemental Figure 25) using the Python package sklearn v.0.22.1 (Pedregosa et al., 2011).

### Analysis of TP N-termini

TP N-termini, defined as the N-terminal 15 amino acids, were analyzed (Supplemental Figure 23) as follows. Charge profiles were generated as described in the literature (Chotewutmontri et al., 2012). The hydrophobicity of TP N-termini was estimated using the HeliQuest standalone application (Gautier et al., 2008). To evaluate disorder, we used IUPred2A, a software that calculates the probability for each residue of being in a disordered region (Erdős and Dosztányi, 2020). We used the default “Long” setting, which has been reported to be more accurate than the alternative “Short” setting (Nielsen and Mulder, 2019). The disorder of a given sequence was taken as the mean of the probability values for each residue (average residue disorder probability).

### Statistical prediction

To evaluate the predictive power of ACC terms (Garrido et al., 2020) obtained for 15-residue TP N-termini with regard to localization (Supplemental Figure 23), we used a binomial logistic regression classifier. We performed 100 5-fold cross-validation runs. In each set of 100 runs, we randomly selected sequences so that the same numbers of mTPs and cTPs were used. For *C. reinhardtii*, we used 33 mTP and 33 cTP sequences, and for *A. thaliana*, we used 29 mTP and 29 cTP sequences. We used an elastic net penalty in a saga solver with an l1-ratio (a parameter) of 0, which is equivalent to using only a ridge penalty, where all features take part in the model, and a C parameter (1/λ) of 0.1. The *a* and 1/λ parameters were optimized with 10-fold cross validation. First, when 1/λ = 1, the best accuracy (0.82) was obtained with *a* between 0 and 0.09. Second, with *a* = 0, the best accuracy (0.82) was obtained with 1/λ = 0.1. A logistic regression model with an elastic net penalty ratio of 0.15 (scikit-learn Python package v.0.22.1) trained on class I HA-RAMPs (Garrido et al., 2020) and *Chlamydomonas* TPs was used to evaluate how similar potential HA-RAMP candidates are to TPs (Supplemental Table 1). Custom scripts were written in Python (v.3.7.6).

### Interaction site prediction

Values for the Boman index, a quantitative proxy for whether a peptide is more likely to interact with proteins (high values) or lipids (low values), were calculated as described in the literature (Boman, 2003). ANCHOR2 interactivity values, a second proxy for protein interaction potential developed for disordered sequences (Mészáros et al., 2009), were calculated using the IUPred2A standalone application and webserver (Mészáros et al., 2018; Erdős and Dosztányi, 2020). Interaction sites for Hsp70 were predicted as described previously (Ivey et al., 2000) based on experimental affinity values for individual amino acids. Putatively TOC-interacting “FGLK” sites were established by searching for the presence of F and [P or G] and [K or R] and [A or L or V] and the absence of [D and E] within each 8-residue window of a sequence, corresponding to rule 22 by Chotewutmontri et al. (2012) that was recommended by the authors in a personal communication. “FGLK-1” sites were established the same way but requiring the presence of only three of the four positive determinants. Custom scripts were implemented in R using RStudio.

### Statistical analysis

*Chlamydomonas* TP distributions (Figure 6 and Supplemental Figure 26) were compared using two-sided *t*-tests (*n* = 34 for mTPs and *n* = 85 for cTPs), and associated mt-set and cp-set HA-RAMPs were compared using one-sided *t*-tests based on the trends set by TPs (*n =* 3 for mt-set and *n* = 5 for cp-set HA-RAMPs) in R using RStudio. Multiple Kruskal statistical tests were performed (same *Chlamydomonas* mTPs and cTPs as above, plus *n =* 382 HA-RAMPs) to evaluate the distribution of amino acids (Figure 3C and Supplemental Figure 21) in the different groups, followed by Dunn post hoc tests (scipy v.1.4.1).

## Data availability

All data are available in the main text or the supplemental materials. Custom code generated in the course of this project will be made available without restrictions upon request to the authors. All plasmids and one independent insertion line per construct (except RP constructs) are available through the Chlamydomonas Resource Centre (https://www.chlamycollection.org/).

## Funding

The following financial support is gratefully acknowledged: the 10.13039/501100004794Centre National de la Recherche Scientifique and 10.13039/501100019125Sorbonne University for annual funding to UMR7141; the Agence National de la Recherche for (a) the “ChloroMitoRAMP” ANR grant (ANR-19-CE13-0009) and (b) “LabEx Dynamo” (ANR-LABX-011), which provided postdoctoral support to O.D.C.; (c) the “MATHTEST” grant (ANR-18-CE13-0027), which provided doctoral support to C.G.; and finally the Fondation Edmond Rothschild, which provided complementary financial support to O.D.C. and C.G. The funders had no role in the design of the study; in the collection, analyses, or interpretation of data; in the writing of the manuscript; or in the decision to publish the results.

## Author contributions

F.-A.W., I.L., and Y.C. conceptualized the project and acquired funds. O.D.C. devised, conducted, and visualized the wet lab experimental part of the investigation and was chiefly responsible for assigning subcellular localization interpretations based on microscopic evidence. C.G. carried out the dry lab/bioinformatic part of the investigation and associated visualization under the supervision of I.L., writing custom software to do so (O.D.C. contributed to the analysis of FGLK and Hsp70-binding sites). C.O.L. developed the custom ImageJ macros used for automated image segmentation. O.D.C. wrote the original draft of the manuscript. F.-A.W., I.L., and O.D.C. reviewed and edited the manuscript. All authors approved the final manuscript.

## Supplemental information

Data S1. Supplemental Tables 1–3

Document S2. Article plus supplemental information
